# Utility of an Emergency Department Observation Unit in Providing Care for Patients With Blunt Thoracic Trauma

**DOI:** 10.7759/cureus.39447

**Published:** 2023-05-24

**Authors:** Shehzad Muhamed, Matthew Vassy, Jason Konzelmann, Jesse Gibson, Leigh Pack

**Affiliations:** 1 Emergency Medicine, Northeast Georgia Medical Center Gainesville, Gainesville, USA; 2 Trauma and Acute Care Surgery, Northeast Georgia Medical Center Gainesville, Gainesville, USA

**Keywords:** emergency medicine and trauma, sternal fracture, rib fractures, trauma management, blunt thoracic trauma, emergency observation unit, emergency department, acute trauma care

## Abstract

Background

The use of Emergency Department Observation Units (EDOUs) to treat patients with a variety of complaints has grown over recent years. However, the treatment of patients with traumatic injuries in EDOUs is infrequently described. Our study sought to describe the feasibility of treating patients with blunt thoracic trauma in an EDOU in consultation with our trauma and acute care surgery (TACS) team. Together, our Emergency Department (ED) and TACS teams designed a protocol for the treatment of patients with specific blunt thoracic injuries (fewer than three rib fractures, nondisplaced sternal fractures) that we felt would require less than 24 hours of care in a hospital setting.

Methods

This study is an IRB-approved retrospective analysis comparing two groups before (pre-EDOU) and after (EDOU) the creation of the EDOU protocol, which was implemented in August 2020. Data was collected at a single, Level 1 trauma center with approximately 95,000 annual visits. Similar inclusion and exclusion criteria were used to select patients in both groups. We conducted two-sample t-tests and Chi-square testing to assess for significance. Primary outcomes include length of stay and bounce-back rate.

Results

A total of 81 patients were included in our data set across both groups. Forty-three patients were included in our pre-EDOU group while 38 patients were treated in our EDOU once the protocol was implemented. Patients in both groups were of similar age, gender and had similar Injury Severity Scores (ISS) ranging from 9 to 14. Hospital length of stay was shorter for the EDOU group (31.5 hours) compared to the pre-EDOU group (36.4 hours) although not statistically significant. When risk stratified by ISS, hospital length of stay did reach statistical significance and was found to be shorter for patients with ISS scores greater than or equal to 9 that were treated in the EDOU (29.1 hours vs. 43.8 hours, p = .028). Both groups had one patient each bounce back for repeat evaluation and additional care.

Conclusion

This study demonstrates the potential use of EDOUs to treat patients with mild to moderate blunt thoracic injuries. The availability of trauma surgeons for consultation along with ED provider experience may be rate-limiting steps in utilizing observation units to care for trauma patients. Additional research with more participants is needed to determine the impact of implementing such a practice at other institutions.

## Introduction

Blunt thoracic trauma comprises an estimated 15% of all traumatic injuries, and patients with chest wall injuries make up approximately 19% of admitted traumas at Northeast Georgia Medical Center. Rib fractures account for many of these injuries, and admission and treatment decisions are based on the number of ribs fractured, fracture patterns, underlying injuries to lung tissue, and anticipated clinical needs [1].

Extensive literature has explored treatment modalities for blunt thoracic injuries. While much of this literature focuses on the appropriate care of patients with significant chest trauma admitted to intensive care with substantial risk for respiratory decline [1,2], many also include recommendations for the treatment of patients with fewer rib fractures. These recommendations focus primarily on early and timely pain management, early mobility, and appropriate monitoring and interventions to prevent complications from respiratory deterioration [3]. Definitive practice management guidelines co-authored by the Eastern Association for the Surgery of Trauma (EAST) and the Trauma Anesthesiology Society have also proposed the utilization of multimodal analgesia for blunt chest trauma, including enteral nonsteroidal anti-inflammatory drugs and oral opioids as needed [4].

These interventions can be delivered for patients with minimal chest trauma in an observation setting. Emergency Department Observation Units (EDOUs) have grown in utility over the last two decades [5]. Multiple studies have demonstrated their benefits, including shorter lengths of stay, lower costs, and higher patient satisfaction [6-7]. EDOUs can be used to provide care for a subset of patients who routinely get admitted for less than 24 hours, and observation unit care can result in decreased length of stay and lower costs. Their use by ED and acute trauma care providers can help offset the demands of trauma and acute care services that are often overburdened with higher patient volumes.

However, the use of EDOUs in the care of trauma patients with minimal thoracic injuries has rarely been described, although they represent a unique set of patients who could be treated in an observation unit and discharged home [8]. Inadequate pain control in patients with blunt thoracic trauma is often the reason cited for the admission of these patients to inpatient care.

## Materials and methods

This study was an institutional review board (IRB) approved retrospective analysis comparing two treatment groups before (pre-EDOU) and after (EDOU) the creation of the Blunt Thoracic Injury EDOU protocol. Our Emergency Department (ED) and Trauma and Acute Care Surgery (TACS) teams collaborated to develop a protocol to treat blunt thoracic injuries in our EDOU, addressing care for patients primarily with fewer than three rib fractures or nondisplaced sternal fractures, stable vital signs, and an absence of significant associated injuries.

The protocol is an order set focusing primarily on multimodal pain management, continuous monitoring of pulse oximetry, aggressive pulmonary toilet and mobility. Patients are admitted to the EDOU by ED physicians with a trauma service consult. Their care is continuously monitored during their stay and patients who then require additional or more intensive management can be admitted by the TACS service to an inpatient unit.

Data was collected over a two-year time frame for both the pre-EDOU (August 2018 to August 2020) and EDOU (September 2020 to September 2022) groups. Patients included presented to a suburban Level 1 trauma center with approximately 95,000 ED visits annually. Our observation unit has a capacity of 24 beds with an annual volume of approximately 7,000 patients. Primary outcomes included length of stay and bounce-back rate.

Similar inclusion and exclusion criteria were used to identify patients within each group. Half of our sample size underwent data validation. Patients with signs of hemodynamic instability, positive imaging findings (e.g., pneumothorax, pulmonary contusion, wide mediastinum, pleural effusion, any vascular injury), requiring intravenous narcotics for pain control or those unable to ambulate were excluded from both groups as these patients would not have met criteria for observation in the EDOU.

We performed two-sample t-tests and Chi-square tests to assess for significant differences between both groups. A one-way ANOVA was performed to compare length of stay for patients with an Injury Severity Score greater than or equal to 9.

## Results

Study data for trauma patients diagnosed with blunt thoracic trauma treated from August 2018 to September 2022 were analyzed to calculate the impact of this observation protocol on length of stay and complication rates, identify characteristics of patients treated in the EDOU at higher risk of complications or clinical decline, and determine the rate of return ED visits and subsequent inpatient admission related to the patient’s thoracic injuries.

Our data set included a total of 81 patients across two groups. Thirty-eight patients were treated for blunt thoracic trauma in the EDOU while 43 patients, who would have met the criteria for placement into the observation unit, were treated as inpatients by the TACS team before the creation of the EDOU (pre-EDOU). Patients in both groups were of similar age (55 years old (SD = 19.5) vs. 61 years old (SD = 13.8), p = .1056 ), gender (58% male vs. 56% male; X^2^ = .035, p = .8513), and had similar Injury Severity Scores (6.9 (SD = 3.6) vs. 6.7 (SD = 2.8), p = .142). Table 1 shows the mechanisms of injury while Table 2 shows the types of injuries treated within each group.

**Table 1:** Mechanisms of injury treated within both groups MVC = motor vehicle collision, MCC = motorcycle collision, EDOU = emergency department observation unit

Mechanism of Injury	Pre-EDOU	EDOU
MVC	25	21
Fall	13	12
MCC	4	4
Bicycle	1	1

**Table 2:** Types of injuries treated within both groups Other = chest wall contusion, EDOU = emergency department observation unit

Injury Type	Pre-EDOU	EDOU
1 Rib Fracture	3	5
2 Rib Fractures	24	18
Sternal Fracture	16	7
Other	0	8

**Figure 1:**
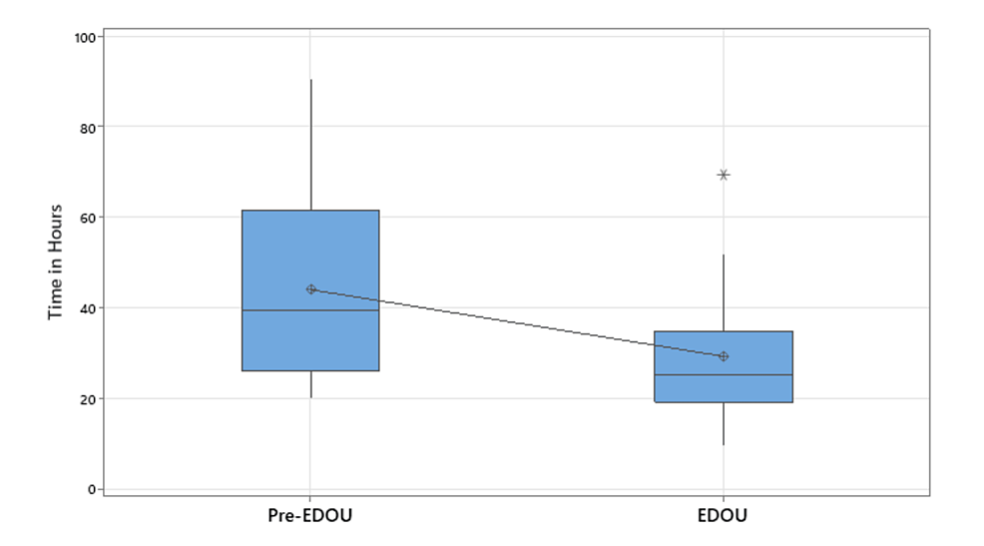
Hospital length of stay for patients with an injury severity score greater than or equal to 9 EDOU = emergency department observation unit

Emergency Department length of stay was similar for both EDOU and pre-EDOU groups (6.7 hours (SD = 4.6) vs. 6 hours (SD = 3.1), p = .4286). Hospital length of stay was shorter for the EDOU group although not statistically significant (31.5 hours (SD = 28.3) vs. 36.4 hours (SD = 18.5), p = .3572). As depicted in Figure 1, hospital length of stay was shorter for the EDOU group for patients with an Injury Severity Score (ISS) greater than or equal to 9 (29.1 hours (SD = 15.1) vs. 43.8 hours (SD = 21.2), p =.028). Both groups had one patient each bounce back to the emergency department for repeat evaluation.

## Discussion

Utilizing EDOUs to care for patients with varying pathologies continues to increase across hospital systems throughout the country. However, the use of EDOUs to care for trauma patients remains rare. Conrad et al. first described the use of observation units to care for trauma patients in 1985. Their study included patients with several types of trauma including closed-head injuries, orthopedic injuries as well as blunt abdominal and thoracic traumatic injuries [9]. Other recent studies have also described care for trauma patients in observation units, but broadly without a focus on a specific mechanism or type of injury. Madsen et al. reported their findings related to care for all types of trauma patients with closed head injury as the most frequent diagnosis [10]. More recently, Holly et al. described the efficacy of an observation unit protocol for trauma patients but included patients with both blunt and penetrating injuries [11]. Both studies reported similar inpatient admission rates from the observation unit (11.5% and 10.4%).

We found only two recent studies that described trauma care in an observation unit specifically for blunt trauma. One study reported on the prevalence of intra-abdominal injury (IAI) in patients presenting with blunt abdominal trauma following ED evaluation and a brief observation period in an EDOU [12]. A separate study assessed the efficacy of EDOUs in treating patients with blunt thoracic trauma at high risk for pulmonary complications [13]. Our goal was to demonstrate that EDOUs can be used to safely care for patients specifically with mild to moderate blunt thoracic injuries without adverse outcomes and few bounce-backs.

Our analysis demonstrates some benefits for treating blunt thoracic injuries in the EDOU. No patients treated for mild to moderate blunt thoracic injury in our EDOU required inpatient admission. Hospital length of stay between the two groups was not statistically significant but did show a trend toward the shorter length of stay for the EDOU group by nearly five hours. While not statistically significant, reducing the length of stay by five hours may be clinically significant considering emergency departments frequently operate above capacity with deleterious effects on patient care and must find novel solutions to optimize throughput [14-15]. The lack of statistical significance was likely due to small sample sizes and may reach significance as we continue to care for patients with blunt thoracic injuries in our EDOU.

As mentioned, ISS scores for both groups were similar and the highest score in each group was 14. Sixteen patients with ISS scores ranging from 9 to 14, indicating moderate injury, did have statistically shorter length of stays in the EDOU compared to the 17 patients treated in the pre-EDOU group. No patients with ISS scores greater than 9 treated in the EDOU bounced back for repeat evaluation. The one patient that did return after treatment in the EDOU had an ISS score of 4 with an isolated sternal fracture as the reason for treatment in the observation unit. The patient returned 48 hours later for symptoms related to COVID-19 and unfortunately, eventually expired due to hypoxic respiratory failure.

Co-management of patients treated in the EDOU for blunt thoracic injuries is an integral part of our treatment protocol. While the EDOU is managed by the emergency department, trauma and acute care surgery sees these patients in consultation and performs tertiary exams before a disposition is made. Within our current system, these tertiary examinations are performed the following day and may be contributing to longer than expected EDOU length of stay greater than 24 hours. We may be able to shorten the length of stay for our patients by performing tertiary exams twice a day and disposition patients that are ready for discharge instead of waiting until the next day.

This study has several limitations to consider. First, the data was collected from a single study site that is a Level 1 trauma center with trauma and acute surgery available at all times. Second, the number of patients included in our data set is small. Lastly, throughput issues related to the COVID-19 pandemic and staffing challenges may have impacted our results as well. Future research should assess the use of EDOUs to care for patients with blunt thoracic injuries across multiple sites including community settings, include more patients and within a time period set apart from micro and macro extraneous factors that may impact throughput.

## Conclusions

The use of EDOUs to care for patients with mild to moderate blunt thoracic injuries should be considered, particularly at institutions that serve as trauma centers. The decision to care for these patients in other settings should be based on the availability of a trauma surgeon for consultation as well as emergency physician comfort and experience.

## References

[1] Unsworth A, Curtis K, Asha SE (2015). Treatments for blunt chest trauma and their impact on patient outcomes and health service delivery. Scand J Trauma Resusc Emerg Med.

[2] Peek J, Smeeing DP, Hietbrink F, Houwert RM, Marsman M, de Jong MB (2019). Comparison of analgesic interventions for traumatic rib fractures: a systematic review and meta-analysis. Eur J Trauma Emerg Surg.

[3] Dennis BM, Bellister SA, Guillamondegui OD (2017). Thoracic trauma. Surg Clin North Am.

[4] Galvagno SM Jr, Smith CE, Varon AJ (2016). Pain management for blunt thoracic trauma: a joint practice management guideline from the Eastern Association for the Surgery of Trauma and Trauma Anesthesiology Society. J Trauma Acute Care Surg.

[5] Wiler JL, Ross MA, Ginde AA (2011). National study of emergency department observation services. Acad Emerg Med.

[6] Perry M, Franks N, Pitts SR, Moran TP, Osborne A, Peterson D, Ross MA (2021). The impact of emergency department observation units on a health system. Am J Emerg Med.

[7] Ross MA, Hockenberry JM, Mutter R, Barrett M, Wheatley M, Pitts SR (2013). Protocol-driven emergency department observation units offer savings, shorter stays, and reduced admissions. Health Aff (Millwood).

[8] Dalla Vecchia C, McDermott C, O'Keeffe F, Ramiah V, Breslin T (2022). Implementation of a chest injury pathway in the emergency department. BMJ Open Qual.

[9] Conrad L, Markovchick V, Mitchiner J (1985). The role of an emergency department observation unit in the management of trauma patients. J Emerg Med.

[10] Madsen TE, Bledsoe JR, Bossart PJ (2009). Observation unit admission as an alternative to inpatient admission for trauma activation patients. Emerg Med J.

[11] Holly J, Bledsoe J, Black K (2012). Prospective evaluation of an ED observation unit protocol for trauma activation patients. Am J Emerg Med.

[12] Kendall JL, Kestler AM, Whitaker KT, Adkisson MM, Haukoos JS (2011). Blunt abdominal trauma patients are at very low risk for intra-abdominal injury after emergency department observation. West J Emerg Med.

[13] Menditto VG, Gabrielli B, Marcosignori M (2012). A management of blunt thoracic trauma in an emergency department observation unit: pre-post observational study. J Trauma Acute Care Surg.

[14] Sartini M, Carbone A, Demartini A (2022). Overcrowding in emergency department: causes, consequences, and solutions-A narrative review. Healthcare (Basel).

[15] Savioli G, Ceresa IF, Gri N (2022). Emergency department overcrowding: understanding the factors to find corresponding solutions. J Pers Med.

